# Identification of sugars and phenolic compounds in honey powders with the use of GC–MS, FTIR spectroscopy, and X-ray diffraction

**DOI:** 10.1038/s41598-020-73306-7

**Published:** 2020-10-01

**Authors:** Katarzyna Kozłowicz, Renata Różyło, Bożena Gładyszewska, Arkadiusz Matwijczuk, Grzegorz Gładyszewski, Dariusz Chocyk, Katarzyna Samborska, Jolanta Piekut, Marzena Smolewska

**Affiliations:** 1 Department of Biological Bases of Food and Feed Technologies, University of Life Sciences in Lublin, Głęboka Str. 28, 20-612 Lublin, Poland; 2 Department of Food Engineering and Machines, University of Life Sciences in Lublin, Głęboka Str. 28, 20-612 Lublin, Poland; 3 Department of Biophysics, University of Life Sciences, Akademicka 13, 20-950 Lublin, Poland; 4 Department of Applied Physics, Lublin University of Technology, Nadbystrzycka 38, 20-618 Lublin, Poland; 5 Department of Food Engineering and Process Management, Institute of Food Sciences, Warsaw University of Life Sciences – SGGW, Nowoursynowska Str. 159C, 02-776 Warsaw, Poland; 6 Department of Agri-Food Engineering and Environmental Management, Białystok University of Technology, Wiejska Str. 45E, 15-351 Białystok, Poland; 7 Faculty Chemical Laboratory, Białystok University of Technology, Wiejska Str. 45E, 15-351 Białystok, Poland

**Keywords:** Biophysics, Biotechnology, Chemical biology, Plant sciences, Materials science

## Abstract

This work aimed at the chemical and structural characterization of powders obtained from chestnut flower honey (HFCh) and honey with Inca berry (HBlu). Honey powders were obtained by spray drying technique at low temperature (80/50 °C) with dehumidified air. Maltodextrin (DE 15) was used as a covering agent. The isolation and evaluation of phenolic compounds and sugars were done by gas chromatography–mass spectrometry analysis. Scanning electron microscopy, Fourier-transform infrared (FTIR) spectroscopy, and X-ray diffraction were performed to determine the morphology of the studied honey powders. The obtained results showed that the content of simple sugars amounted to 72.4 and 90.2 g × 100 g^−1^ in HFCh and HBlu, respectively. Glucose was found to be the dominant sugar with a concentration of 41.3 and 51.6 g × 100 g^−1^ in HFCh and HBlu, respectively. 3-Phenyllactic acid and ferulic acid were most frequently found in HFCh powder, whereas m-coumaric acid, benzoic acid, and cinnamic acid were the most common in HBlu powder. The largest changes in the FTIR spectra occurred in the following range of wavenumbers: 3335, 1640, and below 930 cm^−1^. The X-ray diffraction profiles revealed wide peaks, suggesting that both honey powders are amorphous and are characterized by a short-range order only.

## Introduction

Honey is an aromatic, sweet, and natural food, which is consumed by people around the world. It mainly comprises sugars, water, and other constituents such as enzymes, amino acids, organic acids, vitamins, minerals, carotenoids, and aromatic substances^1,2^.

Compositional data of 152 samples of stingless bee (*Meliponini*) honey showed that 100 g of this natural product contains 58.0–75.7 g of reducing sugars and 1.1–4.8 g of sucrose. Its moisture content varies from 19.9 to 41.9 g × 100 g^−1^, ash content from 0.01 to 1.18 g × 100 g^−1^, diastase activity from 0.9 to 23.0 DN, and invertase activity from 19.8 to 90.1 IU^3^.

Of all sugars found in honey, monosaccharides are the main ones making up about 75%, while the share of disaccharides is 10–15%. Other sugars are also detected in small amounts^4^. According to de La Fuente, et al.^5^ the following sugars can be determined in honeys: fructose, glucose, sucrose, rhamnose, trehalose, nigerobiose, isomaltose, maltose, maltotetraose, maltotriose, maltulose, melezitose, melibiose, nigerose, palatinose, raffinose, and erlose. Other authors including Kaškoniene et al.^6^ identified similar carbohydrates as follows: fructose, glucose, sucrose, maltose, isomaltose, turanose, trehalose, palatinose, cellobiose, raffinose, and panose in all tested samples. In their study, the content of fructose, glucose, and sucrose varied from 329.2 to 400.0 mg g^−1^, from 346.0 to 426.3 mg g^−1^, and from 0.7 to 2.5 mg g^−1^ of honey, respectively.

Following are the primary vitamins present in honey: riboflavin, pantothenic acid, niacin, thiamin, vitamin B6, and ascorbic acid. Honeys also contain minerals such as potassium, sulfur, chlorine, calcium, phosphorus, magnesium, sodium, iron, copper, and manganese^7^. According to some reports, potassium and sodium are the most abundant minerals in honey, and the ratio of potassium to sodium is greater than 1^8^. The composition of minerals and trace elements varies depending on the type of honey. A similar conclusion was drawn by other authors who reported that based on the type the quantities of potassium in honey varied from 298.60 to 491.40 ppm, magnesium from 80.70 to 199.30 ppm, calcium from 60.75 to 99.95 ppm, phosphorus from 21.10 to 33.29 ppm, sodium from 15.69 to 26.93 ppm, iron from 67.18 to 98.13 ppm, iodine from 12.61 to 94.68 ppm, manganese from 4.15 to 6.04 ppm, and zinc from 3.44 to 5.72 ppm.

A large variety of volatile compounds was detected in different honeys, with the knowledge of their sensory and aroma profiles contributing to the characterization of their geographical and floral identity^9^.

Many authors confirmed that honeys are rich in flavonoids and phenolic acids that exhibit a wide range of biological effects and act as natural antioxidants^1,10^. Da Silva et al.^11^ showed that the total phenolic content ranged from 17 to 66 mg GAE g^−1^ of the extract, and the samples with a higher content of phenolic compounds showed higher antioxidant activity.

Da Silva et al.^2^ studied the stability of sugars, proteins, amino acids, enzymes, organic acids, vitamins, minerals, and phenolic and volatile compounds in honey during heating or prolonged storage. The authors determined that the stability of these compounds in relation to the chemical reactions occurring in honey during the process of heating or prolonged storage may compromise its quality; therefore, it is necessary to study the properties of honey after processing.

Powdered honey is an attractive substitute for liquid honey. In order to obtain this product^12^, various drying methods such as spray drying^13–17^, vacuum drying^18–20^, and microwave-vacuum drying^21^ have been proposed so far. Due to the economic effects of the process, most research focuses on spray drying.

In recent works, honey powders have been obtained from multifloral honey, rapeseed honey, and buckwheat honey^13,14,22,23^.

Tests have been conducted on honey powders to analyze their physical properties such as particle shape and size distribution, water content, density, and hygroscopicity^14,15,23^ and evaluate water activity, flowability, cohesiveness, and color. Morphology of the powders was observed using a scanning electron microscope (SEM)^15,23^. Using DCS apparatus, the glass transition temperature of the powders was measured. Moreover, phenolic compounds, antioxidant activity and aroma compounds, diastase activity, and hydroxymethylfurfural content were all investigated^14,22,23^.

Because honey powders are innovative products, very few works comprehensively describe their properties. Therefore, our work aimed at the chemical and structural characterization of powders from chestnut flower honey (HFCh) and honey with Inca berry (HBlu). We used Fourier transform infrared (FTIR) spectroscopy to measure the infrared spectra of honey powders. This method is currently gaining popularity because of its speed, noninvasiveness, and above all, reliability of the results^24,25^. We also examined the structure of honey powders by X-ray diffraction, in addition to microscopic analysis by scanning electron microscopy. Gas chromatography–mass spectrometry (GC–MS) analysis was used for the separation and detection of components like sugars and phenolic acids.

## Materials and methods

### Materials

The research material comprised two honey powders obtained from the chestnut flower honey (*Miele di Sicilia di Prima Sebastiano, Sycylia Włochy*) (HFCh) and honey with Inca berry (*Pasieka Bartnik, Puszcza Białowieska, Poland*) (HBlu). Natural HFCh had an amber color, an intense aroma, and a bitter taste. HBlu comprised 85% of natural honey and 15% of Inca berry.

### Solution preparation and spray drying

Honeys were mixed with water and maltodextrin (MD) DE 15 (PEPEES, *Łomża, Poland*) to obtain a 60% (w/w) solution in which the ratio of honey solids to MD solids was 75:25. Portions containing 400 g of feed solutions were spray dried in a pilot plant spray drier (Niro Minor, GEA) under with the following conditions: feed ratio speed, 0.25 cm^3^ s^−1^; atomization speed, 24,000 rpm; inlet/outlet air temperature, 80/50 °C. Such low drying temperature was achieved by applying additional force of evaporation by air dehumidification, using a dehumidification system comprising TAEevo TECH020 chilling unit (MTA, *Italy*) and condensation–adsorption unit ML270 (MUNTERS, *Sweden*), as described before^22^. During the process of drying, the humidity of the air entering the spray drier was not higher than 1.2 g m^−3^. Powders were packed in plastic (BOPA/PE, 55 μm) bags (Pakmar, *Garwolin, Poland*) and sealed and stored at 25 °C/50% relative humidity.

### Chemical properties of powders

#### Extraction and derivatization of phenolic compounds and sugars

The phenolic compounds were isolated by solid-phase extraction (SPE)^10,26^. For this, 2 g of powdered honey was dissolved in 25 mL acidified water (HCl, pH 2), and the prepared solutions were transferred to the conditioned SPE columns filled with C18 stationary phase (6 mL, 500 mg, Chromabond, Macherey Nagel). Then, the columns were washed with 40 mL deionized water, and sugars and other more polar compounds were eluted. The adsorbed phenolic compounds were eluted with 3 × 5 mL portions of methanol. The collected eluent was dried over anhydrous sodium sulfate and then evaporated to dryness on a rotary evaporator under reduced pressure^27,28^. The extracted dry residue was derivatized with 100 µL BSTFA with 1% TMCS for GC derivatization, Supelco) [N,O-bis(trimethylsilyl) trifluoroactamide with 1% trimethylchlorosilane (for GC derivatization, Supelco)] and 200 µL pyridine (anhydrous, 99.8%, Sigma-Aldrich), and the content was heated at 60 °C for 1 h. TMS [Trimethylsilyl] derivatives were subjected to GC–MS analysis.

Sugars were isolated by using liquid–solid extraction. Briefly, 0.5 g of the sample was mixed with 20 mL of methanol, and then ultrasound-assisted extraction was performed at 40 °C. The obtained extract was dried over anhydrous sodium sulfate and then evaporated to dryness on a rotary evaporator under reduced pressure. Five milligrams of the obtained dry residue was derivatized and analyzed in the same way as samples were analyzed for phenolic compounds.

### GC–MS analysis

The separation and detection of phenolic compounds and sugars were carried out using a 7890B GC System with a 7000C GC/MS Triple Quad mass detector (Agilent Technologies, USA). For the process of separation, the HP-5 ms fused silica capillary column was used (30 m × 0.25 mm × 0.25 μm, Agilent Technologies). Injection temperature was maintained at 260 °C, and the carrier gas flow rate was 1 mL min^−1^ (helium). Temperatures were programmed from 40 to 300 °C at a rate of 3 °C min^−1^ (split 1:10) for the separation of compounds. The detection process was performed in the full scan mode from 45 to 600 m/z. Using the same parameters, all compounds were calibrated.

### Physical properties of powders

#### Water content and water activity

Water content was determined by oven method (105 °C/4 h), while water activity was measured using HygroLab C1 (Rotronic, *Switzerland*) at 25 °C.

#### Microstructure

The outer morphology of powder particles was observed under a scanning electron tabletop microscope TM3000 (Hitachi, *Japan*) operating at 15 kV. Before loading into the SEM chamber, the samples were subjected to metallization (sputtering) with a thin layer of gold and were then observed at a magnification of 500×.

#### FTIR

The infrared spectra of the analyzed samples were measured using 670-IR spectrometer (Agilent, *USA*). To ensure 20-fold internal reflection of the absorbed beam, Attenuated Total Reflectance (ATR) attachment was used in the form of a ZnSe crystal with adequate geometry (truncated at 45°). Sixteen scans were registered during the measurement, and subsequently, the program averaged the results for all spectra. Prior to the measurement, the ZnSe crystal was cleaned using ultraclear solvents (Sigma-Aldrich). Before (1 h) and during the experiment, the measurement chamber was kept in an inert N_2_ atmosphere. Spectral measurements were recorded in the region from 700 to 3800 cm^−1^ at a resolution of 1 cm^−1^. The measurements were taken at the Central Apparatus Laboratory of the University of Life Sciences in Lublin. The spectra were analyzed and processed using Grams/AI software developed by ThermoGalactic Industries (USA). All the spectra were measured at 23 °C.

#### X-ray diffraction

The structure of the powders was studied using Empyrean X-ray diffractometer (PANalytical) with CuKα radiation (λ = 1.54056 Å) and a generator operated at 40 kV and 30 mA. The radiation was detected with a proportional detector. The source divergence and detector slit were 1/2, and Soller slits were applied. The X-ray diffraction profiles were measured in θ−2θ geometry over a range from 10° to 90° with a step of 0.01° and counting time of 5 s per data point at room temperature.

### Statistical analysis

All tests were performed in three replicates, and values were expressed as means ± standard deviations (SDs). Statistical analyses were performed using Statistica software (Statsoft Inc.) at a significance level of *α* = 0.05. The data were analyzed using analysis of variance, and the means were compared using *t*-test.

## Results and discussion

Recently, Samborska et al.^12^ and Jedlińska et al.^29^ have presented a novel approach by the application of dehumidified air for honey spray drying. The additional force of water evaporation provided by the dehumidified air allows using a lower temperature, which, in turn, reduces the amount of carrier. The honey powders thus obtained have an increased quality with a higher content of honey and reduced degradation of biologically active compounds^22^.

Fructose and glucose were identified and quantified in the honey powders, and the results are provided in Table 1. The sum of glucose and fructose contents exceeded the value of 60 g 100 g^−1^ which is required for natural honeys^30^. The contents of simple sugars in HFCh and HBlu were 72.41 and 90.19 g × 100 g^−1^, respectively. The dominant sugar in the powders was glucose (41.34 g × 100 g^−1^ in HFCh and 51.61 g × 100 g^−1^ in HBlu). The total content of disaccharides, considering the share of saccharose, in HFCh and HBlu was 18.03 and 6.34 g × 100 g^−1^, respectively. Juszczak et al.’s^31^ research on herb honeys showed that the content of fructose, glucose, and sucrose ranged from 25.9 to 36.8 g 100^−1^ g, from 23.1 to 33 g × 100 g^−1^, and from 0.4 to 24.8 g × 100 g^−1^, respectively. Significantly a higher content of trisaccharides was found in the HFCh powders.

**Table 1:** Content of sugars in honey powders (g × 100 g^−1^ d.m.).

	HFCh	HBlu		
Total monosaccharides including:	72.41 ± 0.33^a^	90.19 ± 0.13^b^		
Fructose	30.61 ± 0.17^a^	38.10 ± 0.08^b^	y = 2,579,577.8 x − 123,865.8	0.999
Glucose	41.34 ± 0.16^a^	51.61 ± 0.08^b^	y = 2,467,508.8 x − 97,147.7	0.999
Total disaccharides including:	18.03 ± 0.11^b^	6.34 ± 0.08^a^		
Saccharose	0.18 ± 0.01^b^	0.14 ± 0.03^a^	y = 2,935,402 x − 100,325.6	0.998
Total trisaccharides	7.41 ± 0.08^b^	1.68 ± 0.07^a^		
Total carbohydrates	97.85 ± 0.07^a^	98.21 ± 0.13^a^		

Mean values from three repetitions ± SD, Means with different letter in the same row are significantly different (α = 0.05).

Linearity range – 10 – 200 mg/mL (2 – 40 g/kg), limit of quantification – 0.03 mg/mL (0.006 g/kg), limit of detection – 0.01 mg/mL (0.002 g/kg).

Phenolic acids are recognized as health-promoting biological compounds, often referred to as nutraceuticals. Table 2 presents the content of phenolic acids in the analyzed honey powders. On one hand, the highest concentrations of 3-phenyllactic acid (10.478 mg kg^−1^) and ferulic acid (3.110 mg kg^−1^) were found in powders from HFCh. On the other hand, m-coumaric acid (1.979 mg kg^−1^), benzoic acid (1.944 mg kg^−1^), cinnamic acid (1.714 mg kg^−1^), and p-coumaric acid (1.662 mg kg^−1^) were the most common in the case of honey powders with Inca berry. p-Coumaric, ferulic, and syringic acids were identified in the natural honeys^32^. Studies have shown that lime, nectar-honeydew, honeydew, and multiflower honeys were characterized by the highest content of p-coumaric acid (ranging from 290.88 to 677.18 μg 100 g^−1^ honey). Syringic acid was present in nectar-honeydew, honeydew, and buckwheat honeys (ranging from 47.68 to 78.52 μg 100 g^−1^ honey). Profile of phenolic acids, which comprised gallic, chlorogenic, coumaric, caffeic, and syringic acids, was found in all the Australian Eucalyptus honeys^33,34^. Considering the relative composition (%) of the methanol extracts from dry honey (Table 3), 63 different compounds were obtained. These included Β-glucopyranose (19.09%—HFCh; 28.68%—HBlu), β-fructofuranose (18.71%—HFCh; 18.85%—HBlu), and α-glucopyranose (18.67%—the largest share was HFCh; 25.61%—HBlu).

**Table 2:** Content of phenolic acids in honey powders (mg kg^−1^ d.m.).

	**HFCh**	**HBlu**		
Benzoic acid	0.551 ± 0.006^a^	1.944 ± 0.005^b^	y = 92,801.8 x − 23,427.6	0.999
o-Anisic acid	0.004 ± 0.000	–	y = 132,099.9 x − 322,138.3	0.999
m-Anisic acid	0.001 ± 0.000	–	y = 140,223.7 x − 221,502.4	0.998
p-Anisic acid	0.008 ± 0.000^a^	0.030 ± 0.004^b^	y = 130,069.2 x − 303,011.1	0.999
Cinnamic acid	0.064 ± 0.004^a^	1.714 ± 0.004^b^	y = 63,135.6 x − 250,180.9	0.999
2-Nitrobenzoic acid	0.004 ± 0.000^a^	0.004 ± 0.000^a^	y = 78,509.2 x − 295,093.8	0.992
4-Nitrobenzoic acid	0.024 ± 0.004	–	y = 90,620.5 x − 426,501.9	0.994
3-Nitrobenzoic acid	0.005 ± 0.000	–	y = 96,699.4 x − 376,523.9	0.996
3-Phenyllactic acid	10.478 ± 0.007^b^	0.809 ± 0.004^a^	y = 169,112.3 x − 326,913.8	0.999
3-Hydroxyphenylacetic acid	0.029 ± 0.003	–	y = 151,293.4 x − 273,847.1	0.999
4-Hydroxybenzoic acid	0.219 ± 0.005^a^	0.817 ± 0.002^b^	y = 122,618.1 x − 284,143.4	0.999
Vanillic Acid	0.098 ± 0.005^a^	0.730 ± 0.026^b^	y = 34,027.9 x − 64,750.5	0.999
Syringic	0.059 ± 0.006^a^	0.249 ± 0.007^b^	y = 27,070.6 x − 60,122.5	0.999
m-Coumaric acid	0.086 ± 0.002^a^	1.979 ± 0.008^b^	y = 42,592.9 x − 113,937.1	0.999
o-Coumaric acid	0.427 ± 0.004^a^	1.079 ± 0.004^b^	y = 31,973.2 x − 77,293.4	0.998
p-Coumaric acid	0.616 ± 0.006^a^	1.662 ± 0.003^b^	y = 53,359.9 x − 133,220.1	0.998
Ferulic acid	3.110 ± 0.430^b^	0.071 ± 0.002^a^	y = 52,829.8 x − 271,751.4	0.999

Mean values from three repetitions ± SD; means with different letters in the same row are significantly different (*α* = 0.05).

Linearity range: 10–200 mg mL^−1^ (2–40 g kg^−1^); limit of quantification: 0.03 mg mL^−1^ (0.006 g kg^−1^); limit of detection: 0.01 mg mL^−1^ (0.002 g kg^−1^).

**Table 3:** Relative composition (%) of the methanol extracts from dry honey.

Peak	LTPRI	Compound (TMS derivative)	HFCh	HBlu
1	1066	Lactic acid	0.01 ± 0.00	–
2	1081	Glycolic acid	0.01 ± 0.00	–
3	1288	Phosphonic acid	0.02 ± 0.00^a^	0.02 ± 0.00^a^
4	1289	Glycerol	0.03 ± 0.00^b^	0.01 ± 0.00^a^
5	1321	Succinic acid	–	–
6	1509	Malic acid	0.02 ± 0.00	–
7	1641	Ribofuranose	0.01 ± 0.00	–
8	1823	D-Ribonic acid	0.03 ± 0.00	–
9	1836	α-Fructofuranose	8.70 ± 0.20^a^	14.51 ± 0.03^b^
10	1855	β-Fructofuranose	18.71 ± 0.04^a^	18.85 ± 0.00^a^
11	1866	α-Tagatopyranose	0.85 ± 0.04	–
12	1882	α-Talopyranose	–	0.57 ± 0.04
13	1887	β-Fructopyranose	1.61 ± 0.41^a^	2.78 ± 0.04^b^
14	1924	β-Tagatopyranose	–	0.09 ± 0.00
15	1932	α-Glucopyranose	18.67 ± 0.03^a^	25.61 ± 0.07^b^
16	1935	Galactitol	0.04 ± 0.00	–
17	1941	β-D-Galactopyranose	0.03 ± 0.00	–
18	1970	D-Mannitol	0.04 ± 0.00	–
19	1980	D-Glucitol	0.18 ± 0.04^b^	0.02 ± 0.00^a^
20	1996	Inositol	0.18 ± 0.02	–
21	2034	β-Glucopyranose	19.09 ± 0.05^a^	28.68 ± 0.06^b^
22	2036	Palmitic acid	0.04 ± 0.00^b^	0.02 ± 0.00^a^
23	2043	Gluconic acid	1.53 ± 0.04^b^	0.41 ± 0.03^a^
24	2113	Myo-Inositol	0.17 ± 0.03^b^	0.07 ± 0.00^a^
25	2215	(E)-9-Octadecenoic acid	1.02 ± 0.02	–
26	2220	(Z)-9-Octadecenoic acid	0.63 ± 0.03^b^	0.02 ± 0.00^a^
27	2221	α-Linolenic acid	0.97 ± 0.05	–
28	2246	Stearic acid	0.05 ± 0.05	–
29	2418	11-Eicosenoic acid	0.08 ± 0.02	–
30	2420	10-Eicosenoic acid	0.03 ± 0.00	–
31	2449	Eicosanoic acid	0.29 ± 0.05	–
32	2534	Heneicosanoic acid	0.02 ± 0.00	–
33	2558	2-Palmitoylglycerol	0.03 ± 0.00	–
34	2644	Behenic acid	0.53 ± 0.05	–
35	2695	Lactulose	0.03 ± 0.00^a^	0.09 ± 0.00^b^
36	2714	Sucrose	0.14 ± 0.03^a^	0.13 ± 0.02^a^
37	2693	Maltoza, isomer 1	0.25 ± 0.02^b^	0.02 ± 0.00^a^
38	2718	α-Lactose	–	0.10 ± 0.03
39	2758	α-Celobioza	0.84 ± 0.05^b^	0.71 ± 0.03^a^
40	2781	Maltulose, isomer 1	0.91 ± 0.04^b^	0.10 ± 0.01^a^
41	2786	Maltulose, isomer 2	1.84 ± 0.04^b^	0.21 ± 0.02^a^
42	2791	D-Turanoza	2.63 ± 0.04^b^	1.03 ± 0.04^a^
43	2801	Maltose, isomer 2	2.83 ± 0.03^b^	1.76 ± 0.02^a^
44	2811	Isomaltulose, isomer 1	1.11 ± 0.04^b^	0.61 ± 0.03^a^
45	2814	Kojibiose	–	0.02 ± 0.00
46	2816	Trehalose	1.37 ± 0.08	–
47	2835	Isomaltulose, isomer 2	0.89 ± 0.05^b^	0.02 ± 0.00^a^
48	2857	Laminaribiose	1.34 ± 0.04^b^	0.63 ± 0.05^a^
49	2871	β-Cellobioza	0.64 ± 0.05^b^	0.21 ± 0.04^a^
50	2950	Melibiose	0.79 ± 0.07^b^	0.14 ± 0.05^a^
51	2952	a-Isomaltose	0.03 ± 0.00^a^	0.26 ± 0.04^b^
52	2990	β-Gencibiose	1.54 ± 0.04	–
53	3150	Benzo[ghi]perylene	0.10 ± 0.01	–
54	3215	Dibenzo[def,mno]chrysene	0.17 ± 0.03	–
55	3265	unidentified PAH	0.11 ± 0.03	–
56	3342	b-Sitosterol	0.24 ± 0.05	–
57	3462	Melizitose—isomer 1	0.92 ± 0.04	–
58	3504	Raffinose	0.09 ± 0.00	–
59	3517	Kestose	0.99 ± 0.05	–
60	3508	Dibenzo[fg,op]naphthacene	0.15 ± 0.04	–
61	3550	Erlose	0.20 ± 0.03^b^	0.05 ± 0.00^a^
62	3582	Melizitose—isomer 2	4.31 ± 0.03	–
63	3627	Maltotriose	1.90 ± 0.04^a^	2.24 ± 0.04^b^

Mean values from three repetitions ± SD, Means with different letter in the same row are significantly different (α = 0.05).

### Water content and water activity

The obtained honey powders contained, respectively, 3.1 ± 0.1% (HFCh) and 2.8 ± 0.1% (HBlu) of water, while water activity was estimated as 0.196 ± 0.001 (HFCh) and 0.193 ± 0.004 (HBlu). These values are typical for powders obtained by spray drying, both at high temperature applied in a traditional approach^17,35^ and with the use of dehumidified air in a novel approach^12,29^. Low water content (below 4%) and water activity (below 0.2) confirmed the proper conditions for water evaporation created in the drying chamber by applying low temperature and low humidity for processing air.

### Microstructure of powders

**Figure 1:**
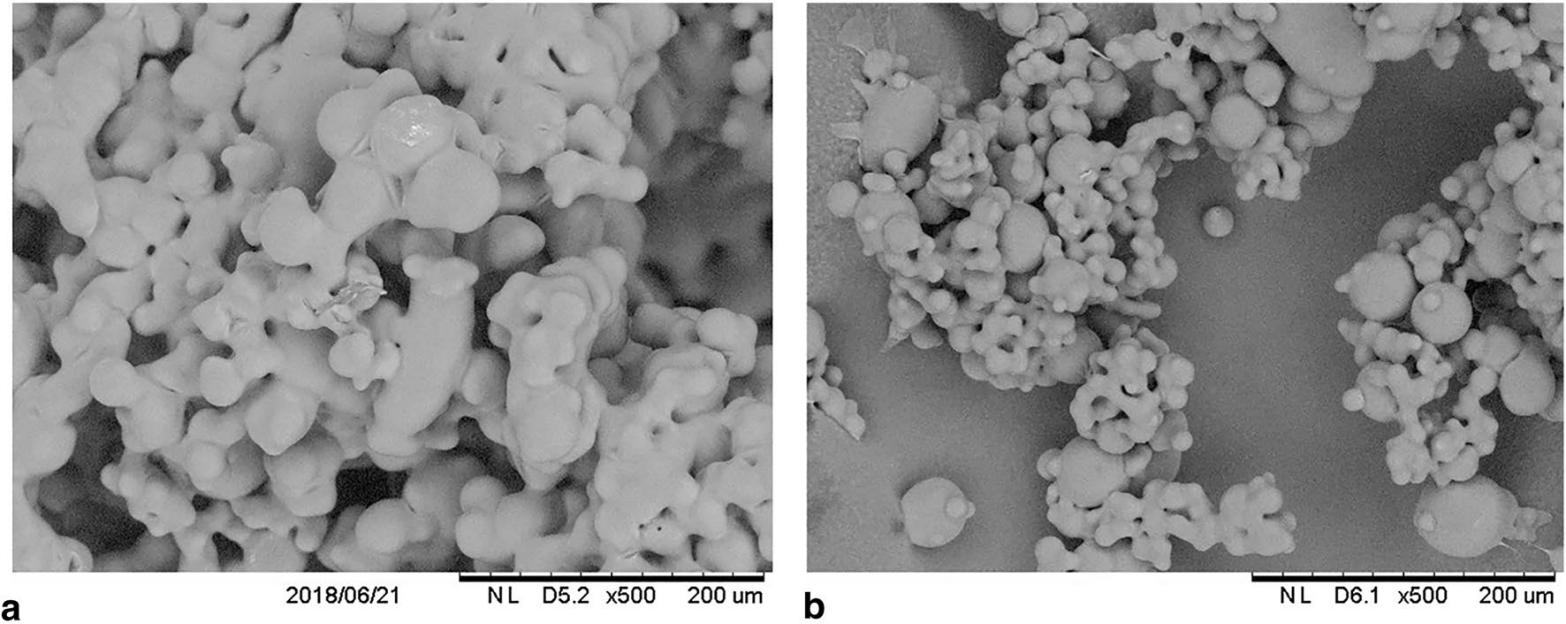
Microstructure of (**a**) HFCh and (**b**) HBlu powders at a magnification of 500 × .

Figure 1 shows the external microstructure of the particles of honey powders with a smooth surface and the linkages between the individual particles, which is very common in the case of honey powders containing more than 70% of honey solids. Spherical particles with smooth surface indicate that the tested honey powders had fully amorphous morphology. Samborska et al.^12^ and Jedlińska et al.^29^ reported a similar morphology for powders containing 80% of honey solids.

### FTIR

**Figure 2:**
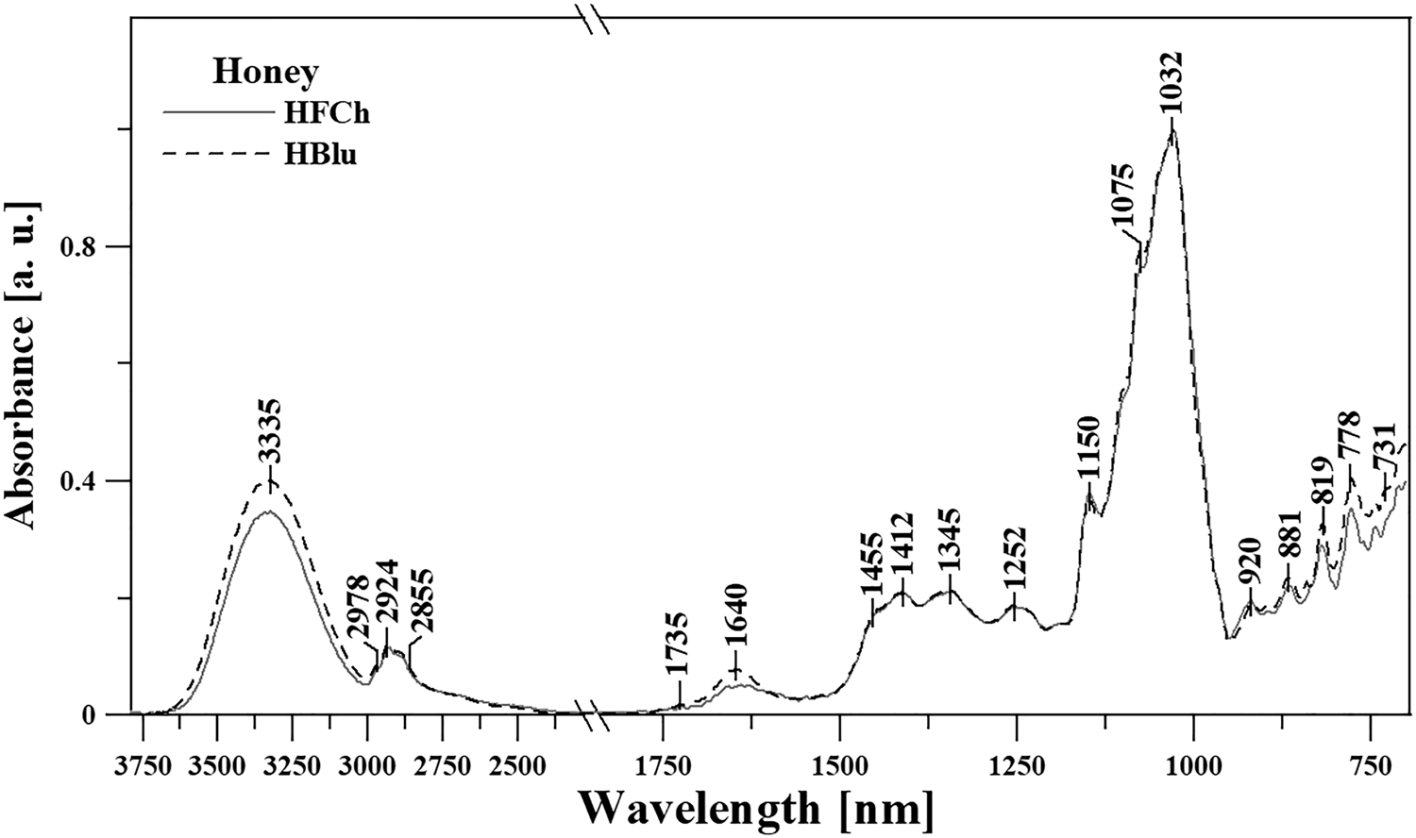
ATR-FTIR spectra of the honey varieties selected for testing, presented in the spectral range from 3800 to 700 cm^−1^. For clarity and ease of presentation, the spectra were normalized for the wave number of 1032 cm^−1^.

In the next stage of the study, ATR-FTIR spectroscopy was used for analyzing in detail the characteristics of the tested HFCh and HBlu powders. Figure 2 and Table 4 (in the spectral range of 3800–700 cm^−1^) present the spectra of the HFCh and HBlu honey samples, which facilitate the correct interpretation and easier characterization of individual bands. Table 4 also assigns bands to the corresponding vibrations of the functional groups in the identified compounds.

**Table 4:** The location of the maxima of absorption bands FTIR with arrangement of appropriate vibration for HBlu and HFCh samples made in terms of spectral 3750–690 cm^−1^.

FTIR	Type and origin of vibrations
Position of bands [cm^−1^]	
**HBlu** and **HFCh**	
3332	ν_st_ (O–H) in H_2_O
2975	ν_s and as_ (C–H) in CH_2_ and CH_3_ group
2916	
2856	
2712	ν (NH_3_) of free amino acids
1734	ν (C = O)
1649	δ_st_ (O–H) in H_2_O
1592	δ (–O–CH) and δ (–C–C–H)
1455	
1410	δ_st_ (O–H) in C–OH group + δ (C–H) in the alkenes
1348	δ (–OH) in C–OH group
1255	ν (C–H) in carbohydrates or/and ν (C–O) in carbohydrates
1193	
1145	ν (C–H) in carbohydrates
1105	ν (C–O) in C–O–C group
1082	ν_st_ (C–O) in C–OH group or ν_st_ (C–C) in the carbohydrate structure, δ (C–H)
1039	
986	
965	
923	ν (C–C) in the carbohydrate structure, δ (C–H)
878	
862	anomeric region of carbohydrates or δ (C–H) (mainly in the structure of sugar)
819	
776	
754	
727	

ν—stretching vibrations, δ—deformation vibrations, s—symmetric, as—asymmetric, st—strong.

According to Anjos et al.^36^ and Svečnjak et al.^37^ the first spectral area ranging from 3650 to about 3000 cm^−1^ (for all samples, Table 4 and Fig. 2), characterized by clear bands with a maximum of about 3335 cm^−1^, corresponds to the stretching vibration of the –OH group of carbohydrates, water, and organic acids. This area is very often attributed to the stretching vibrations of carboxylic acids and also to the –NH_3_ stretching band of free amino acids, which cause a slight strengthening of this area. Other bands, ranging from 3000 to 2800 cm^−1^, correspond to the stretching vibrations of the C–H groups (both alkyl and aromatic, which belong to the sugar backbone). These vibrations belong to the functional groups –CH_2_ and –CH_3_ (alternately symmetrical and asymmetrical). Vibrations with a maximum of ~ 3335 cm^−1^ may also originate from carboxylic acids, the irregular absorption of which (with a wide band coming from the vibrations of the –OH group) significantly increases the C–H stretching vibrations in the systems of the –CH_2_ and –CH_3_ groups.

Due to the formation of strong hydrogen bonds, which in this case belong to carboxylic acid dimers^36^, a wide range of vibrations originate from the ν(–OH) groups. A very clear band with a maximum at about ~ 1640 cm^−1^ (Fig. 2) corresponds, in turn, to the deformation vibrations of the –OH groups. Attention should be paid to a very important area, which is only slightly marked in our spectra (with a maximum at about 1735 cm^−1^) and is due to the stretching vibrations of functional groups such as ketone C = O of fructose and aldehyde CH = O of glucose. It can be seen that it only mildly enhances the vibration with a maximum at 1640 cm^−1^.

A very characteristic area of the samples selected for testing is the fingerprint region (extending from ~ 1480 to 700 cm^−1^, in our case). This region is rich in bands and provides good information about changes in samples occurring due to the use of appropriate factors. The most important vibrations in this area are mentioned (as reported in the literature) as follows: stretching vibrations of C–O, C–C, and C–H and bending vibrations of C–H present in the chemical structure of carbohydrates^36,38^ (often also belong to organic acids and carotenes). The most intense and interesting vibrations from this area are shown by the bands at 1455, 1412, 1345, and 1252 cm^−1^, which mainly come from the deformation of the O–CH group as well as C–C–H in the carbohydrate structure. They can also be strengthened by the deformation vibrations of the –OH groups (with C–OH). The significant area of the bands is in the range of 1244–950 cm^−1^, which are the most intense stretching vibrations in the given samples belonging to the C–H groups, as well as C–O in the carbohydrate structure. The bands at 1150 and 1032 cm^−1^ belong to the vibrations of the C–O and C–O–C groups (Fig. 2 and Table 4). The area from about 1040 to 930 cm^−1^ and below can be significantly strengthened by the stretching vibrations of C–O in C–OH group and stretching of C–C in the carbohydrate structure^37,39,40^. The area below 930 cm^−1^ (from 930 to about 700 cm^−1^) is the vibration area, which is very characteristic for vibrations from the anomeric region of carbohydrates or deformation vibrations of C–H and C–C^24,40^. Even small changes in vibrations from this region usually indicate strong modifications/differences in the sugar fraction bonds (glycosidic bonds). In the case of honey varieties selected for testing, the largest changes occurred at the following wave numbers: 3335, 1640, and below 930 cm^−1^.

### X‐ray diffraction

**Figure 3:**
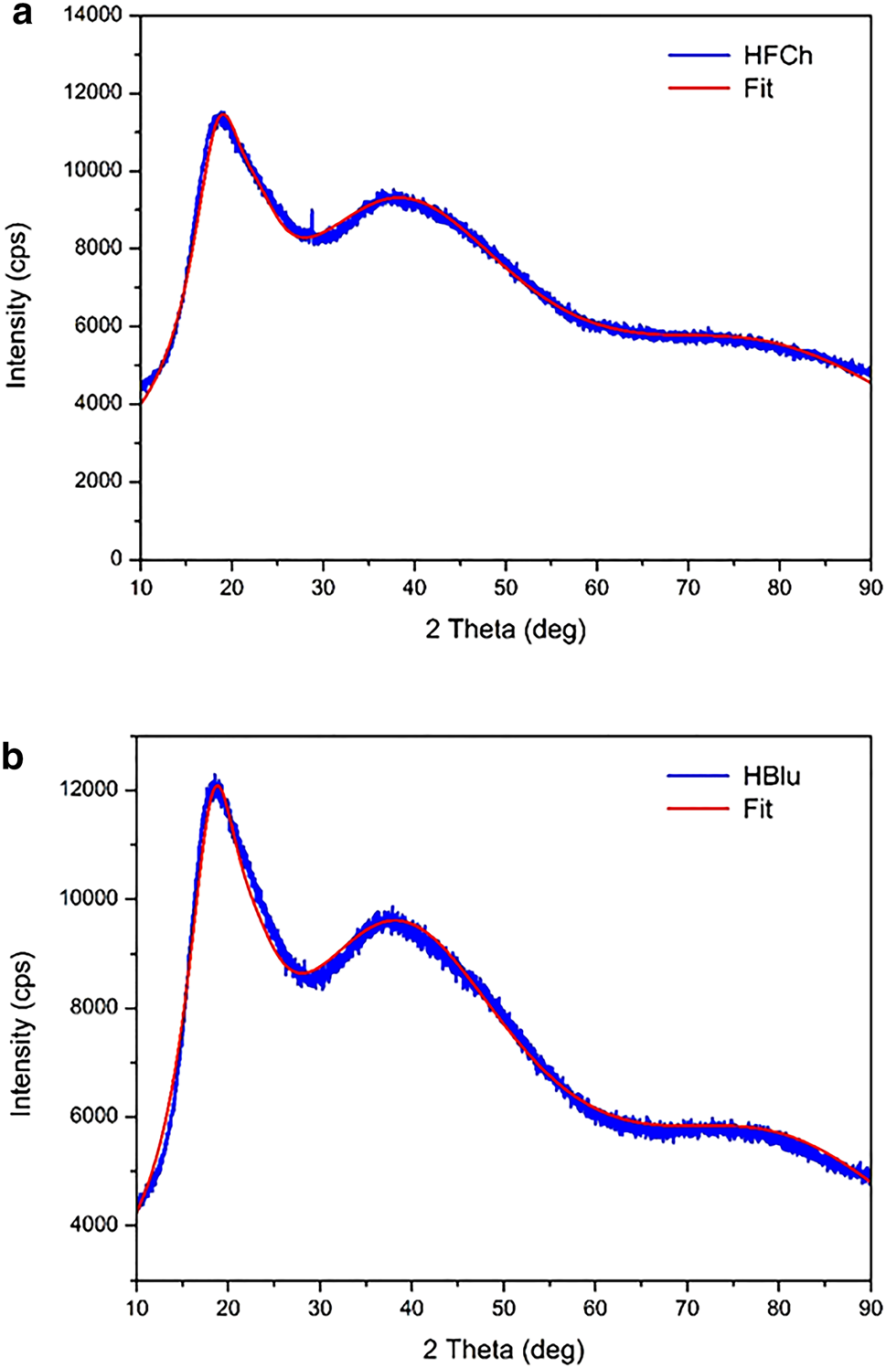
X-ray diffraction profiles (blue) and fit line (red) of (**a**) HFCh and (**b**) HBlu powders.

Figure 3 shows the X-ray diffraction profiles of HFCh and HBlu powders. It can be seen that instead of narrow Bragg peaks, the X-ray diffraction profiles reveal wide peaks, suggesting that the tested honey powders are amorphous and are characterized only by a short-range order. It means that the X-ray scattering is coherent for a small volume and is incoherently averaged over the whole sample. The mean response represents the average local order in the sample. The total amorphous X-ray diffraction profile can be treated as a sum of the Gaussian components^41^ parameterized by their position, amplitude, and SD.

There is no periodic arrangement of atoms and molecules in amorphous materials. However, according to the Gaussian distribution, there are still average characteristic distances between the atoms and molecules located in the material. Because of the normal distribution of these distances, the intensity profiles also have the Gaussian shape. The presence of more than one wide diffraction peak in diffraction profiles indicates that there are a few characteristic distances between atoms or molecules. In our case, it was necessary to assume four components to achieve a good fit of the measured profiles.

Using four Gaussian components, the experimental profiles for both samples were fitted, and the results are presented together with the profiles in Fig. 3. For the HFCh sample, the Gaussian components were present at the scattering angles 2θ = 18.56°, 22.96°, 34.04°, and 79.41°, which corresponded to the distances 4.78, 3.87, 2.36, and 1.21 Å, respectively. However, for the HBlu sample, the Gaussian components were present at the scattering angles 2θ = 18.39°, 22.92°, 38.04°, and 80.67°, and therefore corresponded to the distances 4.82, 3.88, 2.36, and 1.19 Å, respectively. These distances determine the average distances between atoms in molecules. The amplitudes of the Gaussian components were in the ratio 3.81:1:4.71:2.56 for HFCh sample and 4.24:1:5.08:2.72 for HBlu sample. For individual Gaussian components, SDs of 3.65°, 3.68°, 14.4°, and 21.13° were obtained for both HFCh and the HBlu samples. The results indicate that both powders have a very similar structure characterized by a short-range order only.

## Conclusion

The results of the study showed that the content of simple sugar was 72.4 g × 100 g^−1^ in HFCh and 90.2 g × 100 g^−1^ in HBlu. Glucose was the dominant sugar at an amount of 41.3 g × 100 g^−1^ in HFCh and 51.6 g × 100 g^−1^ in HBlu. The concentrations of total disaccharides were equal to 18 g × 100 g^−1^ in HFCh and 6.3 g × 100 g^−1^ in HBlu. 3-Phenyllactic acid and ferulic acid were most frequently found in HFCh powders, whereas m-coumaric acid, benzoic acid, cinnamic acid, and p-coumaric acid were the most common acids in the case of HBlu. The largest changes in the FTIR spectra occurred in the range of the following wavenumbers: 3335, 1640, and below 930 cm^−1^. FTIR offers unique advantages, as it reflects the overall vibrations of the components and their interactions within the samples as spectra; it is also shown to be a reliable method to quantify the majority of the sugar content in honey and is easily adapted to the routine analysis of this product. The microstructure analysis and X-ray diffraction profiles revealed wide peaks, suggesting that the honey powders are amorphous and are characterized by a short-range order only. The results of the Gaussian components indicated that both samples have a very similar structure. The use of a novel spray drying method allowed obtaining honey powders that retained their health-promoting properties. These honey powders are an innovative product with potentially wide applications in the food industry.

## Data Availability

All the data generated or analyzed during this study are included in this published article (and its Supplementary Information files).
